# Dietary fenugreek seed extract improves dry matter intake, apparent total tract nutrient digestibility, and alters whole blood transcriptome of Holstein dairy heifers

**DOI:** 10.1093/tas/txac132

**Published:** 2022-09-25

**Authors:** Godstime Taiwo, Taylor Sidney, Modoluwamu Idowu, Francisca Eichie, Theodore P Karnezos, Ibukun M Ogunade

**Affiliations:** Division of Animal and Nutritional Science, West Virginia University, Morgantown, WV 26505, USA; Division of Animal and Nutritional Science, West Virginia University, Morgantown, WV 26505, USA; Division of Animal and Nutritional Science, West Virginia University, Morgantown, WV 26505, USA; Division of Animal and Nutritional Science, West Virginia University, Morgantown, WV 26505, USA; Purina Animal Nutrition, Arden Hill, Arden Hills, MN 55126, USA; Division of Animal and Nutritional Science, West Virginia University, Morgantown, WV 26505, USA

**Keywords:** animal health, phytobiotics, saponin

## Abstract

This study was conducted to evaluate the effects of dietary supplementation of a fenugreek seed extract (**SAP**) as a source of saponins on dry matter intake, blood metabolites, apparent total tract nutrient digestibility, and whole blood transcriptome of Holstein dairy heifers. Eight heifers (**BW** = 477 ± 23.8 kg) were stratified by BW and then randomly assigned to one of two treatments in a cross-over design with two 35-d experimental periods and a 14-d wash-out between the two periods. The heifers were housed individually in eight dry lot pens. Each pen was equipped with one GrowSafe intake node. Treatments were 1) corn silage-based diet with no additive (**CON**) and 2) corn silage-based diet plus 2 g per hd per d of SAP. Dairy heifers fed supplemental SAP had higher (*P* ≤ 0.05) DMI and apparent total tract digestibility of dry matter, crude protein, and neutral detergent fiber compared to CON. Dairy heifers fed supplemental SAP had lower (*P* = 0.03) blood urea nitrogen and higher (*P* = 0.01) blood glucose concentration compared to CON. Pathway analysis via gene set enrichment analysis revealed increased (FDR ≤ 0.05) transcript levels for gene sets belonging to ISG15 antiviral mechanism, metabolism of proteins, citric acid cycle and respiratory electron transport, ATP synthesis by chemiosmotic coupling, and complex I biogenesis in dairy heifers fed supplemental SAP compared to CON. Decreased (FDR ≤ 0.05) transcript levels for gene sets associated with erythrocytes take up/release carbon dioxide, release/take up oxygen, and O_2_/CO_2_ exchange in erythrocytes were also observed with SAP supplemental group. Taken together, our results revealed that fenugreek seed extract can be used as an effective dietary supplement for dairy heifers to improve intake and digestibility, and alter the host transcriptome toward improved energy and amino acid metabolism, improved antiviral immune status, and reduced oxidative stress damage.

## INTRODUCTION

The sub-therapeutic use of antibiotics in animal feed as growth promoters has come under scrutiny in recent years due to human health hazards associated with antibiotic resistance (de Lange et al., 2010; Heo et al., 2013). Therefore, research efforts have been geared toward developing and evaluating a wide range of feed additives including phytobiotics as alternatives to in-feed antibiotics (Benchaar et al., 2008; Kamra et al., 2008). Phytobiotics contain several phytochemicals (including saponins and other polyphenolic compounds) with vast animal health benefits including antimicrobial, antiviral, antioxidant, and immune-stimulating effects (Davidson and Naidu, 2000; Hossain et al., 2015). In fact, saponins have been demonstrated in several research studies to modulate rumen microbial population and fermentation, with a consequent positive effect on overall animal performance and health (Patra and Saxena, 2009; Anantasook et al., 2015; Huws et al., 2018). Seeds of Fenugreek (*Trigonella foenum-graecum* L.), an annual leguminous plant that is available in several parts of the world, contain steroidal saponins as the most abundant phytochemical (Kaviarasan et al., 2007). Although Yucca and Quillaja saponins have been used as feed additives and are currently available as commercial products (Cheeke and Oleszek, 2006), the sources (plant types and plant parts) influence the biological activities of saponin (Hassan et al., 2010). Therefore, studies are needed to evaluate other sources of saponin on nutrient utilization and health status of animals. Many studies have reported the positive effects of fenugreek seed as a source of saponins in humans, rats, and pigs (Chevallier, 1996; Hossain et al., 2015), however, its effects on performance, health, and blood transcriptome of ruminants have not been extensively investigated. Transcriptome analysis of biofluids in ruminants provides a robust insight into health and metabolism (Jiminez et al., 2021; Taiwo et al., 2022b). Therefore, the objective of this study was to determine the effects of dietary supplementation of fenugreek seed extract as a source of saponins on performance, blood metabolites, apparent total tract nutrient digestibility, and whole blood transcriptome of Holstein dairy heifers.

## MATERIALS AND METHODS

All animal experimental procedures were reviewed and approved by West Virginia University Animal Care and Use Committee (protocol number: 2104041214).

### Animals, Housing, and Treatments

A total of eight Holstein dairy heifers (BW = 477 ± 23.8 kg; age = 402 ± 13 d) were stratified by BW and then randomly assigned to one of two treatments in a cross-over design with two 35-d experimental periods and a 14-d wash-out between the two periods at the West Virginia University Agricultural Research Farm. The heifers were housed individually in eight dry lot pens. Each pen is equipped with one GrowSafe intake node (GrowSafe Systems Ltd., Airdrie, Alberta, Canada). Treatments were 1) corn silage-based diet with no additive (**CON**) and 2) corn silage-based diet plus 2 g per hd per d of fenugreek seed extract (**SAP**). The CON diet was fed to all the dairy heifers regardless of dietary treatment group during the 14-d wash-out period. The basal diet, fed as a TMR (Table 1), was supplied ad libitum daily at 0800 hours to achieve at least 5% ort. Fenugreek seed extract was provided by Purina Animal Nutrition (Purina Animal Nutrition, Arden Hills, MN) and was reported to contain 19,500 mg/kg sapogenin. The fenugreek seed extract was weighed into gelatin capsules and dosed orally (one capsule daily for 32 d) to each dairy heifer before the TMR was fed in the morning to ensure complete consumption of the additive.

**Table 1.** Ingredient and nutrient composition of the basal diet^*^

Item	Value†
Ingredient composition	
Corn silage	37.0
Chopped triticale straw	30.0
Concentrate supplement‡	33.0
Nutrient composition‖	
DM, %	44.5
CP	15.3
aNDF	59.1
ADF	30.4
EE	3.45
Ca	0.66
P	0.37
NE_m_, Mcal/kg	1.46
NE_g_, Mcal/kg	0.99

Composition of basal diet calculated from analysis and concentration of individual ingredients.

Values are presented on a % DM basis unless indicated otherwise.

Jump Start supplement (Southern States Cooperative, Richmond, VA) contained processed grain by-products, yeast culture, ground limestone, urea, salt, cane molasses, potassium sulfate, magnesium sulfate, sodium selenite, vitamin A supplement, calcium carbonate, vegetable oil, manganous oxide, vitamin D3 supplement, vitamin E supplement, zinc oxide, lecithin, phosphoric acid, basic copper chloride, magnesium chloride, propylene glycol, natural and artificial flavors, ferrous sulfate, calcium iodate, and cobalt carbonate; guaranteed analysis: 14% CP; 1.3% Ca; 0.40% P; 1.0% Na; 1.75% salt; 0.8% K; and 6,600 IU/kg vitamin A.

DM, dry matter; CP, crude protein; aNDF, neutral detergent fiber (amylase treated); ADF, acid detergent fiber; EE, ether extract; NE_m_, net energy of maintenance; NE_g_, net energy of gain.

### Dry Matter Intake

The quantity (as fed) of feed consumed by each heifer was recorded daily using a GrowSafe system. Daily samples of diets offered were sampled and dried in a forced-air oven at 52 °C for 48 h to determine daily DMI. Samples of feed ingredients were collected once every 5 d, dried for 48 h at 52 °C in a forced-air oven, and ground using a 1-mm screen (Wiley Mill; Arthur H. Thomas Co.). The samples were then pooled for each period and sent to a commercial laboratory (Dairy One Forage Laboratory, Ithaca, NY) for analysis of N (method 990.03; AOAC International, 2000), NDF (Van Soest et al., 1991), and ADF (method 989.03; AOAC International, 2000).

### Apparent Total Tract Digestibility Measurements

On days 30 to 35 of each experimental period, daily TMR and fecal samples were collected for analysis of indigestible neutral detergent fiber (**iNDF**) as the digestibility marker (Cole et al., 2011) to determine the apparent total tract digestibility of nutrients (DM, CP, NDF, and ADF). The TMR samples were collected immediately after feeding and stored at −20 °C. Fecal samples (approximately 20 g) were collected from each heifer two times daily at 1200 and 1600 hours directly from the ground immediately after defecation and then stored at −20 °C. At the end of the experiment, TMR and fecal samples were dried at 60 °C for 48 h in a forced-air oven and then ground through a 2-mm screen using a Wiley mill (Arthur H. Thomas Co., Philadelphia, PA). After drying and grinding, the samples were pooled for each heifer in each period. Thereafter, the samples were sent to a commercial laboratory (Dairy One Forage Laboratory, Ithaca, NY) for analysis of DM, NDF, ADF, CP, and indigestible NDF. Total feces output (kg) was calculated as iNDF consumed (g/d) divided by fecal iNDF concentration (g/kg; Cole et al., 2011). Total tract digestibility was then calculated based on nutrient intake and fecal nutrient output.

### Blood Sample Collection

Before morning feeding (at 0700 hours), blood samples from the heifers were collected via jugular venipuncture in 3-mL blood tubes containing sodium heparin (BD Vacutainer, Franklin Lakes, NJ) before morning feeding on days 0, 7, 14, 21, 28, and 35 of each experimental period. Immediately after collection, a sub-sample of the whole blood (750 µL) was transferred into lithium heparin cup and immediately analyzed using a Custom Idexx Chemistry panel (IDEXX BioAnalytics, Columbia, MO) for total protein, glucose, blood urea nitrogen, albumin, creatinine, total cholesterol, amylase, lipase, total bilirubin, aspartate aminotransferase (**AST**), alanine aminotransferase (**ALT**), and gamma-glutamyl transferase (**GGT**). Another sub-sample of the whole blood (500 µL each) collected on day 35 of each experimental period was immediately transferred into RNA-protect tubes (cat. no. 76554; Qiagen) and stored at −80 °C for subsequent RNA extraction and sequencing.

#### RNA extraction and sequencing.

 Total RNA was extracted from whole blood samples using RNeasy Protect Animal Blood kit (Catalog No. 73224; Qiagen) according to the manufacturer’s protocol. RNA concentration integrity number were measured using NanoDrop One C spectrophotometer (Thermo Fisher Scientific, Waltham, MA, USA) and Agilent 2100 Bioanalyzer (Agilent Technologies), respectively. All RNA samples had an A260:A280 ratio from 1.8 to 2.0 and RNA integrity numbers > 8.0. Approximately 100 to 250 ng of total RNA per sample was used for preparation of dual indexed RNA Libraries using the KAPA RNA HyperPrep Kit with RiboErase (Human, Mouse, Rat) Globin Reduction method according to the kit manufacturer’s instructions (Roche Diagnostics, Basel, Switzerland). Library quality was assessed by electrophoretic analysis on the Agilent 4200 TapeStation system with High Sensitivity D1000 screentape. The RNA libraries were then sequenced in a dual indexed 2 × 50 paired-end run on an Illumina NextSeq2000 equipped with a P3 flow (Illumina, San Diego, CA).

### Data and Statistical Analysis

Parameters such as DMI and apparent total tract nutrient digestibility were analyzed as a randomized block design using the GLIMMIX model of SAS (SAS 9.4, SAS Inst. Inc., Cary, NC) using animal as the experimental unit. The model included the fixed effects of treatment, period, and their interaction. Blood metabolites that were measured repeatedly over time were analyzed using repeated measures and tested for the effect of treatment (CON vs. SAP), period, day of collection, and the day × treatment interaction. Day 0 data were used as covariates and appropriate covariance structures were used based on the lowest Akaike values (Wang and Goonewardene, 2004). Normality was tested by examining the distribution of residuals. Differences between means were determined using the Fisher’s test and results were considered significant at *P* ≤ 0.05.

Analysis of the RNA-seq data has been described in our previous study (Taiwo et al., 2022b). Briefly, the removal of low-quality base calls and adapter sequences was performed using Trimmomatic v 0.39 (Bolger et al., 2014), and then the quality-filtered reads were aligned to *Bos_taurus*.ARS-UCD1.2 reference genome using HISAT2 v 2.2.1 (Kim et al., 2015). The resulting files were then sorted and indexed, and PCR and optical duplicate reads were marked using SamTools v1.12 (Li et al., 2009). The numbers of reads in each sample mapping to each gene were computed using the R/Bioconductor package GenomicAlignments v 1.26.0 (Lawrence et al., 2013) and the log_2_ fold change values were computed using DESeq2 version 1.30.1 (Love et al., 2014). Gene set enrichment analysis (**GSEA**) was applied to analyze differentially expressed gene set pathways considering the transcript levels of sets of biologically related genes (Reimand et al., 2019) using the R/Bioconductor package fgsea v 1.16.0. The differentially expressed gene sets were identified using FDR ≤ 0.05.

## RESULTS AND DISCUSSION

Compared to CON, dietary supplementation of SAP increased (*P* ≤ 0.05) the DMI and apparent total tract digestibility of dry matter, crude protein, and neutral detergent fiber of the dairy heifers (Table 2). Though no studies evaluating the effects of SAP on DMI in ruminants are currently available, a previous study reported increased feed intake in Male Wistar rats administered 100 mg fenugreek seed extract per 300 g of BW (Petit et al., 1993). The increased DMI observed in SAP steers compared with CON could be explained by greater apparent digestibility of nutrients. Greater NDF digestibility observed in these animals suggests increased fiber digestibility, which is known to increase ruminal passage rate of feed with consequent increase in feed intake (Dado and Allen, 1996). Consistent with this current study, Goetsch and Owens (1985) observed an improvement in apparent total tract organic matter digestibility in dairy cattle fed supplemental saponin. Hristov et al. (2004b) observed increased in situ ruminal dry matter degradability when a yucca extract (saponin) product was fed to dairy heifers. Similarly, Lu and Jorgensen (1987) reported significant increases in hemicellulose and cellulose digestion associated with inclusion of alfalfa saponins in a concentrate-based diet for sheep. In fact, in a recent study, saponin was reported to induce a shift in both the ruminal microbial community and metabolome toward efficient carbohydrate metabolism (Wang et al., 2019). Although we did not measure rumen microbiome in this study, previous studies have shown that saponin may increase fiber digestion and consequent nutrient utilization in cattle by increasing the relative abundance of some fibrolytic microbes such as *Bacterioidetes, Prevotella_1, and Prevotellaceae_YAB2003* (Emerson and Weimer, 2017; Klevenhusen et al., 2017; Wang et al., 2019).

**Table 2.** Dry matter intake and apparent nutrient digestibility of dairy heifers fed diet supplemented with no or 2 g per hd per d of fenugreek seed extract as a saponin source

	Treatment*		SEM	*P*-values
	CON	SAP		
Dry matter intake, kg/d	7.86	9.10	0.43	0.01
Digestibility (%)				
Dry matter	61.7	65.6	1.04	0.01
Crude protein	58.5	62.2	1.14	0.02
Acid detergent fiber	51.4	52.3	1.09	0.86
Neutral detergent fiber	55.3	58.9	1.51	0.05

CON, control; SAP, diet supplemented with fenugreek plant extract (Purina Animal Nutrition, Arden Hills, MN).

In the current study, dairy heifers fed supplemental SAP had lower (*P* = 0.03) blood urea nitrogen and higher (*P* = 0.01) glucose concentrations compared to CON (Table 3). Blood urea nitrogen is often used as a measure of amino acid metabolic status of ruminants (Hammond, 1983; Bed et al., 1997; Nozad et al., 2012). Consistent with our result, previous studies on some saponin-rich extracts (*Yucca Schidigera* and tea saponin) reported reduced blood urea nitrogen concentration (Wallace et al., 1994; Hristov et al., 1999). Steroidal saponin has been reported to have defaunating effect in the rumen, which suppresses activity of rumen protozoa, thereby reducing amino acid deamination (Wallace et al., 1994; Wina et al., 2005). Reduced amino acid deamination and bacterial proteolysis in the rumen reduce ammonia production and increase the flow of microbial protein and amino acids available for absorption in the intestine (Williams and Coleman, 1991; Chanu et al., 2020). Consistent with this study, Guyader et al., 2017 reported reduced ruminal ammonia concentration which was explained to be due to the toxic effect of tea saponin on protozoa (Wina et al., 2005). Thus, reduced blood urea nitrogen observed in dairy heifers fed supplemental SAP could be attributed to reduced amino acid deamination or increased uptake of ammonia by bacteria to generate microbial protein (Wang et al., 2014).

**Table 3.** Blood metabolites of dairy heifers fed diet supplemented with no or 2 g per hd per d of Fenugreek seed extract as a saponin source

Items	Treatment*		SEM	*P*-value
	CON	SAP		
Glucose, mg/dL	74.0	79.7	0.81	0.01
Creatine, mg/dL	0.76	0.80	0.02	0.38
Phosphorus, mg/dL	7.18	7.44	0.23	0.48
Blood urea N, mg/dL	7.27	5.42	0.51	0.03
Calcium, mg/dL	9.18	9.16	0.14	0.92
Total protein, g/dL	6.83	7.14	0.15	0.27
Albumin, g/dL	3.00	2.93	0.07	0.62
Globulin, g/dL	3.92	4.11	0.17	0.52
ALT, U/L	51.6	48.4	3.09	0.48
ALKP, U/L	120	121	9.60	0.95
GGT, U/L	22.1	27.4	2.48	0.18
Bilirubin, mg/dL	0.44	0.38	0.06	0.54
Cholesterol, mg/dL	149	147	4.69	0.76
Amylase, U/L	65.7	62.4	3.59	0.55
Lipase, U/L	73.5	81.5	7.23	0.53

CON, control; SAP, diet supplemented with fenugreek plant extract (Purina Animal Nutrition, Arden Hills, MN). Treatment*day *P*-value > 0.05.

Blood glucose concentration is regulated by the rate of feed intake (Mayer, 1953, Allen et al., 2005). The increased blood glucose concentration of dairy heifers fed supplemental SAP relative to CON could be attributed to their increased DMI and/or nutrient digestibility. Another possible explanation for increased blood glucose concentration is the activities of several other metabolites contained in SAP. Fenugreek seed extract, in addition to its richness in saponin, also contains several other metabolites such as tannins, alkaloids, flavonoids, terpenoids, and glycosides (Khorshidian et al., 2016; Al-Timimi, 2019). Condensed tannin supplementation has been reported to increase the molar proportion of propionate, a glucogenic precursor formed in the rumen which is expected to increase blood glucose concentration (Makkar et al., 1995). Taken together, the lower blood urea nitrogen and higher blood glucose concentration observed in the current study for the dairy heifers fed supplemental SAP suggests a balanced supply of amino acids and energy-yielding nutrients for optimal health and performance of the dairy heifers (Blummel et al., 1999; Chumpawadee et al., 2005; Gonzalez-Munoz et al., 2019).

In order to assess the effects of SAP supplementation on the overall metabolism of the dairy heifers, we analyzed the whole blood transcriptome via GSEA. Gene expression in blood cells reflects physiological changes in the whole body and possibly indicates biological processes related to overall body metabolisms (Jegou et al., 2016; Taiwo et al., 2022b). The application of pathway analysis based on GSEA provides information and insight into the metabolic processes that are differentially regulated due to dietary supplementation of SAP. In this study, an average of 39 million reads per sample was generated (Supplementary Table S1). Pathway analysis by GSEA demonstrated increased (FDR ≤ 0.05) transcript levels for gene sets belonging to ISG15 antiviral mechanism, metabolism of proteins, citric acid cycle and respiratory electron transport, ATP synthesis by chemiosmotic coupling, and complex I biogenesis in dairy heifers fed supplemental SAP (Table 4). Research studies have shown that saponins and certain sapogenins from some phytogenic extracts are effective at deactivating viruses by initiating a decrease in microsomal enzyme activity (Sindambiwe et al., 1998; Nguyen et al., 2000). Similarly, Li et al. (2015) reported that tea seed plant extract rich in saponin inhibits porcine reproductive and respiratory syndrome virus replication, by inactivating the virus via blockade of the virus entry into the cells or indirectly through the modulation of Poly (A)-binding protein. Our results and those of others that utilized other saponin-rich extracts demonstrated that dairy heifers fed supplemental SAP probably have a better ability than CON to quickly recognize viral pathogens and initiate an antiviral defense.

**Table 4.** Whole blood RNA-seq-based gene set enrichment pathway analysis of dairy heifers fed diet supplemented with no or 2 g per hd per d of Fenugreek seed extract as a saponin source

Pathway	FDR	NES*	Gene set size (number of leading-edge genes)	Leading-edge genes†
ISG15 antiviral mechanism	0.04	Positive	9 (4)	ISG15, MX2, ARIH1, JAK1
Metabolism of proteins	0.01	Positive	250 (91)	ELOB, RPL39, RPS21, UBE2F
Erythrocytes take up carbon dioxide and release oxygen	0.01	Negative	3 (3)	HBB, HBA1, HBA
Erythrocytes take up oxygen and release carbon dioxide	0.01	Negative	3 (3)	HBB, HBA1, HBA
O_2_/CO_2_ exchange in erythrocytes	0.01	Negative	3 (3)	HBB, HBA1, HBA
The citric acid cycle and respiratory electron transport	0.05	Positive	48 (23)	NDUFA7, NDUFA13, NDUFB5, ATP5F1E
ATP synthesis by chemiosmotic coupling	0.03	Positive	42 (22)	NDUFA7, NDUFA13, NDUFB5, ATP5F1E,
Complex 1 biogenesis	0.03	Positive	31 (17)	NDUFA7, NDUFA13, NDUFB5, NDUFB10,
Respiratory electron transport	0.03	Positive	31 (17)	NDUFA7, NDUFA13, NDUFB5, NDUFB10

NES, normalized enrichment score; Positive NES indicates upregulated pathways in dairy heifers fed fenugreek seed extract compared to the control; negative NES indicates downregulated pathways in dairy heifers fed fenugreek seed extract compared to the control.

Leading edge genes are those that are enriched within the gene set. See Supplementary Table S2 for the full list of leading-edge genes.

Increased transcript level for gene sets belonging to metabolism of proteins is an indication of improved amino acid status and is in concordance with reduced blood urea nitrogen observed in dairy heifers fed supplemental SAP. Several studies in ruminants have demonstrated that amino acid metabolism is essential for optimizing performance, health, and immunity against infections and stress (Li et al., 2007, 2015; Taiwo et al., 2022a). Increased transcript levels for gene sets belonging to the citric acid cycle and respiratory electron transport, ATP synthesis by chemiosmotic coupling, and complex I biogenesis in SAP steers support the increased blood glucose concentration and probably indicate improved energy metabolism and synthesis to support health and performance of the animals. Taken together, these results suggest that dietary supplementation of SAP improved amino acid and energy metabolism of the dairy heifers.

Erythrocytes contain hemoglobins that are in continuous exposure to high oxygen content which predisposes them to oxidative stress (Olsson et al., 2007, Taiwo et al., 2022b). Oxidative stress, a consequence of an imbalance of pro-oxidants and antioxidants, can lead to cell and tissue damage, resulting in impaired health and performance of animals (Sordillo and Aitken, 2009; Celi and Gabai, 2015). In this study, we observed decreased (FDR ≤ 0.05) transcript levels for gene sets associated with erythrocytes take up/release carbon dioxide, release/take up oxygen, and O_2_/CO_2_ exchange in erythrocytes in dairy heifers fed supplemental SAP compared to CON, suggesting reduced oxidative stress. Previous studies in humans demonstrated that Fenugreek was effective at reducing oxidative hemolysis in erythrocytes (Rayyan et al., 2010; Belguith-Hadriche et al., 2013). Our result is also consistent with those observed with tea saponins (Yoshiki et al., 1998; Hu et al., 2002). For instance, Wang et al. (2017) observed reduced oxidative stress in dairy cows after 6-wk dietary supplementation of tea saponins. Similarly, some studies have shown that a group of saponins called soyasaponin contain antioxidant moiety (2, 3-dihydro-2, 5-dihydroxy-6-methyl-4H-pyran-4-one), which allows saponin to scavenge superoxides by forming hydroperoxide intermediates, and thus preventing bio-molecular damage by free radicals (Yoshiki et al., 1998; Hu et al., 2002). In addition, Ginseng saponins can induce a superoxide dismutase gene, that encodes one of the major antioxidant enzymes (Kim et al., 1996). Therefore, the decreased transcript level of gene sets related to the erythrocytes in this study is an indication of a strong antioxidant defense system mediated by SAP supplementation.

## CONCLUSION

Dietary supplementation of SAP to dairy heifers increased DMI and apparent total tract digestibility of dry matter, crude protein, and neutral detergent fiber. Dairy heifers fed supplemental SAP had lower blood urea nitrogen and higher blood glucose concentration compared to CON. Results of GSEA of whole blood transcriptome data revealed altered transcript levels of gene sets associated with improved antiviral mechanism, amino acid metabolism, ATP synthesis, and antioxidant defense system in dairy heifers fed supplemental SAP. Further studies are needed to evaluate the effects of supplemental SAP on growth performance and immune response in animals, especially under stress conditions.

## Supplementary Material

txac132_suppl_Supplementary_Table_S1

txac132_suppl_Supplementary_Table_S2
